# Evaluation of various prosthetic materials and newer meshes for hernia repairs

**DOI:** 10.4103/0972-9941.27721

**Published:** 2006-09

**Authors:** H G Doctor

**Affiliations:** Jain Clinic and Bhatia General Hospitals, Mumbai, Grant Medical College and J. J. Hospital, Mumbai, India, ETHICON Institutes of Surgical Education and Johnson and Johnson Medical India

**Keywords:** Hernia repair, mesh, prosthesis

## Abstract

The use of prosthesis has become essential for repair of all hernias since the recurrence rates are consistently lower when they are used. To fulfill this requirement, a variety of newer meshes have been engineered. An ideal prosthesis should be strong, pliable, non-allergenic, inert, non-biodegradable, non-carcinogenic and should stimulate adequate fibroblastic activity for optimum incorporation into the tissues. Prosthesis used for hernia repairs can be non-absorbable, composite (combination of absorbable and non-absorbable fibres) or with an absorbable or a non-absorbable barrier. Surgeons should acquire sufficient knowledge of different types of prosthesis so as to select an appropriate one for a given case. Non-absorbable or composite mesh is recommended for hernia repair where it will not come in contact with the bowel. Prosthesis with a barrier only should be used for intra-abdominal placement to prevent bowel adhesions since it is increasingly difficult to defend the use of a biomaterial that has no adhesion barriers. This review highlghts all these different types of meshes and their appropriate selection for a given hernia repair. Selection of the optimum size and its proper fixation is mandaory. Complications can be avoided or minimized with proper selection of mesh for a given case and by performing the surgery with a meticulous technique.

Abdominal wall hernias may be repaired either by closing the defect under tension with sutures or by reinforcement of the defect with a mesh. Suture techniques, either for primary repair or applied after failure of a primary repair, are characterized by high recurrence rates.[1] The use of mesh has become essential in the repair of all hernias - inguinal, ventral or incisional. Recurrence rates are consistently lower when mesh is used and a variety of meshes have been developed for the purpose.[1] The only exceptions for the use of mesh are pediatric hernias and a contaminated surgical site. Specially designed mesh is mandatory for intra-abdominal placement.

## PHYSIOLOGICAL RECONSTRUCTION

Generally, the task of a surgical mesh implant is to provide biomechanical strength to the attenuated fascial structures. Surgical mesh is designed to withstand the tension forces acting on the abdominal wall. Further, the mesh must not impede and ideally should facilitate the healing process of the hernial defect by encouraging ingrowth of the body's own connective tissue by the induction of strong collagen tissue around the mesh fibers. The advantage that large pore size mesh offers over traditional small-pore mesh is that the tissue is able to grow through the large pores of the mesh and create a thinner, more integrated scar instead of the thicker, less flexible scar that is created with mesh of minimal pore size. Ultimately, this creates a more elastic scar and surrounding tissue for the patient.[2] Further, latest technological advances have allowed the use of proprietary materials to develop several different types of meshes of alternate construction methods that allow adequate support to the abdominal wall while substantially reducing the amount of foreign body material implanted in the patient. This new type of mesh - which has become commonly known as ‘lightweight construction’ or ‘reduced-mass’ mesh - offers a combination of thinner filament size, larger pore size and a percentage of absorbable materials and has the capability of being more closely aligned with the physiologic properties of the abdominal wall.

## COMPARISON OF MESH CONSTRUCTION

The strength of the traditional micro-porous or heavyweight mesh is derived from the use of a large mass of material, which in turn contributes to stiffness, excessive scar plate formation and abdominal wall restriction, leading to reduced patient comfort and chronic pain. Although the reinforcement and strengthening of the abdominal wall to prevent recurrence is the main task of a mesh, functional restrictions can impair quality of life.

According to recent reports, about half of the patients with a large mesh prosthesis within the abdominal wall express complaints such as paresthesia at the palpable stiff edges of the mesh and the physical restriction of abdominal wall mobility. Physical capacity and, therefore, patient quality of life is fundamentally affected by the integrity of the abdominal wall.

Restoring the physiological properties of the abdominal wall must take into account the complex interactions of the anatomic structures and also focus, in particular, on the resulting tensile strength and elasticity.[2]

From this, it may be assumed that the flexibility of the abdominal wall is restricted by implantation of extensive foreign material and to a greater degree by excessive scar tissue formation.[2] In addition, the nonphysiological low stretching capability and relative stiffness of heavyweight mesh materials contrast with the highly elastic abdominal wall and can give rise to shearing forces. These forces favor the formation of weak scar tissue and thus recurrence at the edges of the mesh implant.

## STRENGTH REQUIREMENTS

**Figure 1:**
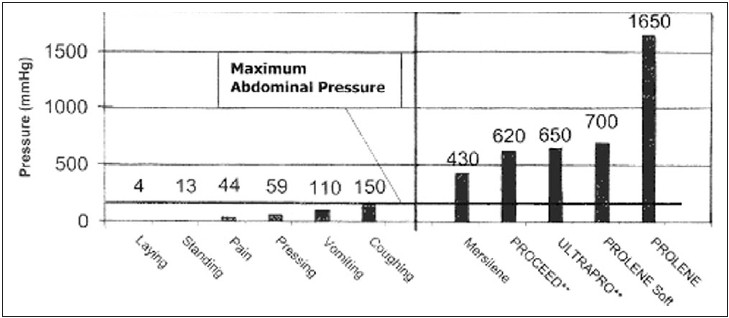
Comparison of abdominal pressure with burst strength

Surgical mesh must also provide sufficient biomechanical strength to meet physiological requirements in order to permanently protect the fascial defect. The tension on the abdominal wall and the required tensile strength of the fascia closure is a function of the intra-abdominal pressure, which ranges from 1.55 mmHg in patients lying supine up to 150 mmHg maximum peak pressure in coughing patients [Figure 1].

Given that the maximum tensile strength of the abdominal wall is 150 mmHg, traditional heavyweight small-pore meshes are shown to be considerably oversized. For example, in order to rupture polypropylene (PROLENE, Johnson and Johnson) mesh, an intra-abdominal pressure of more than 10 times the maximum pressure would be required.[2]

## TYPES OF PROSTHESES

Three prosthetic materials and / or their various combinations are most commonly used today for repair of hernias. They are polypropylene, polyester and ePTFE.

1. Polypropylene mesh is most widely used for the past 20 years because of its stability, strength, inertness and handling qualities. It has stood the test of time. These meshes are made up of polypropylene fibers arranged in a network with pores of differing sizes. The product differs with different manufacturers regarding size of the monofilament material, size of pores, its thickness, pliability and shrinkage. They are known as Marlex (Davol Inc, Cranston, USA), PROLENE (Johnson and Johnson, India), PROLENE Soft (Johnson and Johnson, India) and Surgipro - multifilament (Tyco Healthcare, USA). Monofilament mesh is preferable as it is less likely to give rise to infection.
2. Polyester meshes (Dacron, MERSILENE [Johnson and Johnson, India]) are not very popular with laparoscopic surgeons though they are widely used in France and were used for hernia repairs by Stoppa technique.
3. PTFE meshes are smooth, soft and strong. They allow good tissue ingrowth. They are more expensive.

The first two meshes are ideal for use where they do not come in contact with the abdominal viscera, viz, laparoscopic repairs of inguinal hernias - TAPP or TEP. Though some surgeons use it as intra-abdominal placement for repair of ventral and incisional hernias, this is not advisable since literature reports of complications of bowel adhesions, bowel obstruction, fistulization and erosion into abdominal viscera even after many years. ‘Placement of polypropylene mesh in the abdominal cavity is not a problem for the surgeon placing the mesh, but it can be a disaster for the surgeon who has to do the next operation on that patient.’ - Guy Voeller.[3] Latest technological advances have now made available prosthetic materials which prevent bowel adhesions.

### Lightweight composite meshes without barrier

**Figure 2:**
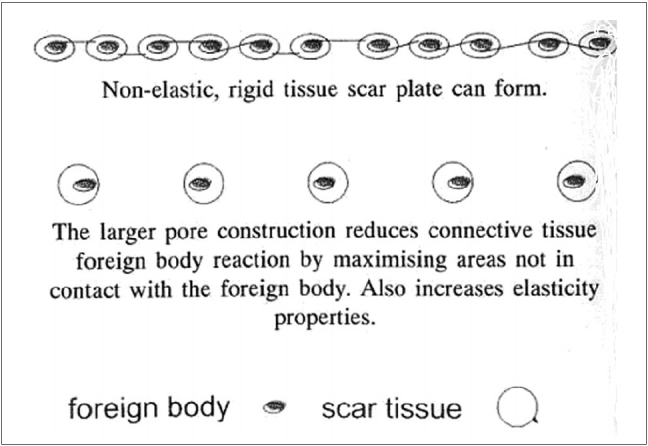
Pore size and scar tissue in lightweight composite meshes

VYPRO II and ULTRAPRO (Johnson and Johnson, India) are meshes specially designed to reinforce weak tissues for open repair of inguinal hernias as well as in TAPP or TEP. They consist of thin filaments of VICRYL and PROLENE (Johnson and Johnson, India) or MONOCRYL (Johnson and Johnson, India) and PROLENE (Johnson and Johnson, India). These filaments are twisted together and then knitted to form a requisite mesh structure. They are partially absorbable since they have 50% VICRYL or MONOCRYL. They are macroporous, the pore size being 4.5 mm and this induces a better tissue ingrowth of a strong three-dimensional collagen fiber network.[2] This construction results in almost 70% reduction of implanted foreign body and results in ‘scar-mesh’ as opposed to ‘scar-plate’ [Figure 2]. These meshes provide enough strength to the tissues and allow optimum mobility to the abdominal wall.

Repair of ventral and incisional hernia is nowadays undertaken by minimal access surgery rather than the conventional open technique. To achieve this repair, intraperitoneal placement of the mesh requires a material which has both high tissue ingrowth towards the abdominal wall and nonadhesiveness on the other side to prevent bowel adhesions.

In an effort to avoid or minimize the possible detrimental effects of macroporous mesh when placed intraperitoneal, composite meshes with absorbable and nonabsorbable barriers were engineered.[4] In all these composite meshes, the layer facing the abdominal cavity prevents adhesions with the bowel while the layer in contact with the abdominal wall encourages high tissue in growth in polyester, polypropylene or ePTFE.

### Absorbable barrier composite meshes

#### Sepramesh:

Sepramesh Biosurgical composite (Genzyme Biosurgery, Cambridge, USA) is a dual-component prosthetic biomaterial composed of macroporous polypropylene on one side, with bioresorbable, nonimmunogenic membrane of sodium hyaluronate and carboxymethyl cellulose on the other side. Seprafilm was designed to provide protection against intra-abdominal adhesion formation throughout the critical period of remesothelialization during the first postoperative week. The absorbable barrier turns to a gel in 48 h, remains on the mesh for ∼7 days and is cleared from the body in 28 days. This antiadhesive material forms a physical barrier on damaged surfaces to prevent adherence or reduce viscosity between opposing tissues. The physical barrier should allow injured tissues to heal separately from each other. In addition, the sodium hyaluronate and carboxymethyl cellulose are anionic polyaccharides that form a membrane that is negatively charged, a molecular property that promotes the separation of healing tissues.

#### Parietex Composite and Parientene Composite meshes:

Parietex composite (Sofradim, France) is composed of multifilament polyester mesh with a purified, oxidized bovine atelocollagen type I coating covered by an absorbable, antiadhesion film of polyethylene glycol and glycerol. Polyethylene glycol is a hydrogel that decreases tissue adherence and glycerol is a hydrophobic lipid. The collagen coating functions to promote collagen ingrowth by increasing the hydrophilicity of the polyester mesh and decreasing the fibrous tissue reaction to the ‘foreign’ material (mesh). The collagen, polyethylene glycol and glycerol film are resorbed in ∼3 weeks.

Parientene composite mesh consists of the same antiadhesive barrier but coated to polypropylene.

#### PROCEED surgical mesh:

**Figure 3:**
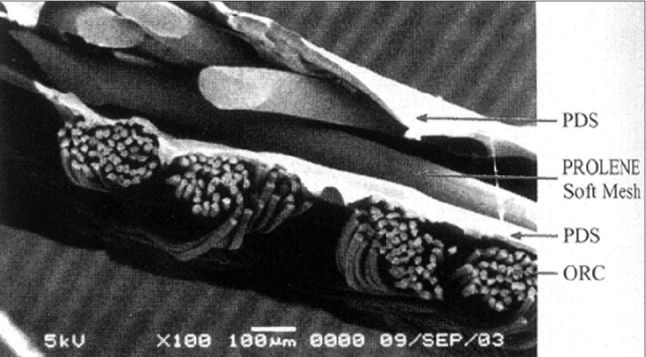
Multilayered structure of a PROCEED surgical mesh

PROCEED surgical mesh (Johnson and Johnson, India) is a sterile multilayered, thin, flexible, laminate mesh comprised of an oxidized regenerated cellulose (ORC) fabric; and PROLENE soft mesh, a nonabsorbable polypropylene mesh which is encapsulated by a polydioxanne polymer. The polypropylene mesh side of the product allows for tissue ingrowth, while the ORC side provides a bioresorbable layer that physically separates the polypropylene mesh from underlying tissue and organ surfaces during the wound-healing period to minimize tissue attachment to the mesh. The polydioxanone provides a bond to the ORC layer [Figure 3].

It has a lightweight macroporous mesh construction, leaves behind less residual foreign body, allows the fluid to flow through easily and does not harbor bacteria.

### Nonabsorbable barrier composite meshes

#### Bard composix mesh (Davol Inc, Cranston, USA):

This is a combination of polypropylene that has a thin coat of ePTFE on one side to prevent bowel adhesions. Introduction through a laparoscopic port is difficult because it cannot compress the mesh and hence requires a larger port (12 mm) for its introduction.

#### GORE-TEX dual mesh:

The Gore-Tex Dual mesh (W. L. Gore, USA) material has two surfaces; one is very smooth (micropores 3 mm) and the other is rough (micropores approximately about 22 mm). It is designed to be implanted with the smooth surface against the visceral organs - tissue to which no or minimal adhesion is desired - and the other surface against which tissue incorporation is desired. The Dual mesh comes in two choices: one is a solid sheet and the other is perforated to allow for greater tissue incorporation. A recent innovation is the incorporation of silver and chlorhexidine into the ePTFE. This results in a significant antimicrobial action.

The use of novel absorbable and nonabsorbable barriers on composite meshes to reduce the incidence of adhesion and adhesion-related complications has been evaluated in animal models and a few clinical studies have been reported. The incidence of adhesions and tenacity of adhesions were reduced for all of the barrier meshes compared with the macroporous polyester mesh.

The development of new prosthetic biomaterials with the addition of absorbable and nonabsorbable barriers for adhesion prevention after intra-abdominal placement of mesh during open and laparoscopic hernia repair is potentially a significant advancement in the management of ventral and incisional hernias. Long-term follow-up is desirable to determine if Sepramesh, Parietex Composite, Parientene Composite, PROCEED surgical, Bard Composix and Gore-Tex Dual meshes will decrease the incidence of mesh-related complications compared to nonbarrier, macroporous meshes.

## NORMAL PERITONEAL HEALING: MESOTHELIALIZATION

The peritoneum is unique in its healing characteristics, histology and cellular biochemistry. The parietal peritoneum is comprised of a single layer of mesothelial cells covering a continuous basement membrane. This overlies loose connective tissue consisting of fibroblasts, collagen fibers, adipocytes, leukocytes, lymphatics and microvasculature. The visceral peritoneum's basement membrane overlies the extracellular matrix of the specific organ.

Healing and regeneration of injured peritoneal mesothelium is unlike that of any other epithelial-like surface. It has been known since 1919 that peritoneal healing differs from that of skin, which heals gradually by epithelialization from the border. Defects in the parietal peritoneum, in contrast, heal by simultaneous epithelialization of the entire surface. Hence complete mesothelialization, developing from multiple points throughout the defect, occurs just as rapidly for large and small defects.[5]

The peritoneum remesothelializes in 5–8 days and if adhesions can be prevented during this critical period, perhaps biomaterial-related complications can be prevented or reduced. This neoperitoneum serves not only as a physical barrier separating the mesh from the abdominal viscera but potentially promotes fibrinolysis through the release of tissue plasminogen activator and inhibition of cell-cell and cell-tissue interaction through the release of hyaluronic acid.[5]

While the severity and extent of adhesions may change over weeks and months, the incidence of adhesions - that is, whether they develop at all - is decided in the first 5–7 days after peritoneal trauma takes place. Development of intraperitoneal adhesions is a dynamic process that actually begins at the time of incision when surgically traumatized tissues in apposition have their first opportunity to bind though fibrin bridges.[6]

The ideal product for the prevention of adhesions should therefore be easy to apply, remain where placed for 7 days, produce no inflammation or foreign body reaction, not impair wound healing or mesothelial cell growth and migration and absorb when no longer needed.[5]

## SELECTION OF MESH

An appropriate mesh should be selected and implanted. For inguinal hernia repair by TAPP or TEP, any of the following meshes can be used:

1. Conventional polypropylene mesh
2. PROLENE soft mesh
3. VYPRO II
4. ULTRAPRO

For intra-abdominal placements, any mesh that will prevent bowel adhesions should be used. It can be either ePTFE, PROCEED surgical mesh or any one of the newly engineered meshes with an absorbable or a nonabsorbable barrier.

Next important matter to consider is the size of the mesh. It must be at least 15 × 15 cm for an inguinal hernia. For repair of umbilical, ventral and incisional hernia, it should be at least 4 cm wider than the defect. It is better to initially measure the size of the defect with the scale and then select a mesh of appropriate size. It should be wide enough to cover the defect in all directions since a smaller size may lead to protrusion of the mesh into the defect and result in a recurrence.

## FIXATION OF MESH

Mesh should be appropriately fixed either with sutures, staples, tackers or Endoanchors (Johnson and Johnson, India). If the mesh is not fixed, it may migrate and cause a recurrence. For intra-abdominal placement of the mesh, a few strong sutures should be placed at least at four corners of the mesh. Subsequently, mesh should be fixed with anchors at a distance of 3 cm all around to prevent any bowel obstruction by getting in between the mesh and the defect.

A fixation device such as a tacker, when used, should not protrude too much beyond the mesh as it can cause adhesion to a bowel loop. Tackers penetrate only 2 mm beyond the mesh and do not provide the same holding strength as sutures. Tensile strength of suture is 2.5 times greater than the tacker. Full thickness abdominal wall suture fixation is necessary to prevent contraction and / or migration of the mesh. Point fixation with only staples or tackers fixes the mesh to the peritoneum only and not directly to the fascia.[4]

**Figure 4:**
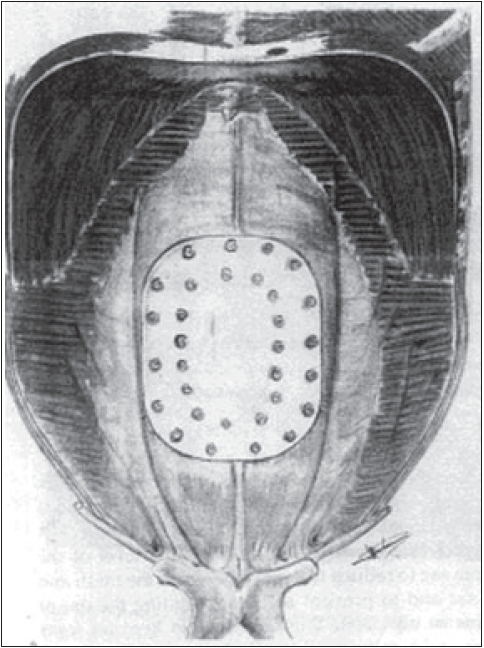
Double crown technique for laparoscopic ventral hernia repair

Alternatively, the double crown technique described by Conde *et al* for placing tackers can be used.[7] This offers a number of advantages over the ‘combined suture and tacker’ method. Tackers are initially placed 1 cm apart at the edge of the mesh and a second inner crown of tackers is placed 1 cm apart at the edge of the defect [Figure 4]. Abdominal wall is pressed down when tacks are applied.

Tissue adhesives like fibrin sealants (FS) and cyanoacrylates may be used to fix the mesh. A randomized prospective trial demonstrated a significant reduction of analgesic requirement by using FS for mesh fixation during bilateral TEP, but it was associated with an increased incidence of postoperative seroma.[8] FS is a safe and efficacious alternative to mechanical stapling for the anchorage of mesh during TEP. It allows biologic fixation of the mesh onto the pelvic floor without the risk of causing neurovascular injury and serves as an additional tool for prosthetic mesh fixation during TEP.

## COMPLICATIONS

These are minimal if cases are properly selected and a meticulous technique is performed.

### Pain

Unlike most laparoscopic procedures, fixation of a mesh by a laparoscopic technique for ventral / incisional hernia repair, especially when it is large, can be very painful initially when the pain is caused by the fixation of the mesh with sutures.[3] Pain resolves over a period of time and then is localized at one or two individual fixation sites. It can be relieved by drugs or by a local injection of an anesthetic agent.

### Seroma

It is often a normal occurrence and resolves over a period. In rare cases, aspiration may be done using sterile technique in the operation theater.[3]

The other complications of laparoscopic repair of hernia include injury to the urinary bladder; bowel adhesions; bowel obstruction; bowel fistulization; erosion into intra-abdominal organs; migration of the mesh and recurrence - when mesh is of a smaller size and when it is not properly fixed.[9]

## SUMMARY

The use of prostheses has become essential for repair of all hernias since the recurrence rates are consistently lower when they are used; and to fulfill this requirement, a variety of novel meshes have been engineered. Surgeons should acquire adequate knowledge of all different types of prostheses in order to select an appropriate one for a given case. Prosthesis with either an absorbable or a nonabsorbable barrier should be used for intra-abdominal placement to prevent bowel adhesions, since it is increasingly difficult to defend the use of a biomaterial that has no adhesion barrier in direct contact with the abdominal viscera. Selection of an optimum size and its proper fixation are mandatory. Complications may be avoided or minimized with proper selection of cases and performance of a meticulous technique.
